# Program evaluation of a virtual physical activity program for individuals with disabilities

**DOI:** 10.3389/fspor.2023.1128565

**Published:** 2023-05-02

**Authors:** Nikki Matthews, Roxanne Seaman, Emily Bremer

**Affiliations:** School of Kinesiology, Acadia University, Wolfville, NS, Canada

**Keywords:** virtual program, adapted physical activity, intervention, physical literacy, physical activity, disability

## Abstract

**Introduction:**

Regular physical activity is important for positive health outcomes yet, most individuals do not meet physical activity guidelines. Recent studies show that one in five Canadians aged 15 or older have one or more disabilities, yet as a population, individuals with disabilities are 16%–62% less likely to meet physical activity guidelines. The COVID-19 pandemic created additional barriers to physical activity participation as lockdowns prevented in-person programming. In response to the pandemic, the Acadia University Sensory Motor Instructional Leadership Experience (S.M.I.L.E.) Program shifted its programming to a virtual platform; however, there was little research to guide its creation, implementation, or expected outcomes. Thus, this program evaluation explored program feasibility and impact on physical activity and physical literacy.

**Method:**

A mixed methods case study approach was used for this project. Virtual S.M.I.L.E. took place over eight weeks in the fall of 2020. Programming consisted of three live Zoom sessions facilitated by trained program leaders, and eight weeks of at-home activity guides for participants to complete on their own time. Demographic data, physical literacy (PLAYself), and physical activity (IPAQ-A) data were collected using caregiver pre-and post-programming surveys. Throughout programming, weekly check-in surveys were sent to reflect on the previous week of programming. After the eight weeks of programming were complete, caregiver and leader interviews were conducted to understand both program implementation and performance perspectives.

**Results:**

Results indicated that participants' (*N *= 15, M_age _= 20.4 years) overall physical literacy and physical activity did not change; however, there was a decrease in the cognitive domain of physical literacy (*p *= 0.03). Caregiver and leader interviews highlighted five main themes following the virtual programming: (a) Virtual impact on programming; (b) Programming impact on social and motor goals; (c) Impact of program design; (d) Impact on physical activity; and (e) Program feasibility for families.

**Discussion:**

Results from this program evaluation suggest that physical literacy and physical activity levels were generally maintained throughout programming and caregivers indicated several social and activity benefits. Future work includes program modifications and further evaluation of virtual adapted physical activity programming to improve the physical literacy of individuals with disabilities.

## 1. Introduction

Physical activity is associated with an abundance of physical, mental (1–4) and social (1, 2, 5) health benefits for all age groups. While the benefits of physical activity have been well documented, the most recent report from Statistics Canada shows that under half of Canadians meet the physical activity guidelines (6). The data for individuals with disabilities is even more concerning with one recent literature review demonstrating that individuals with a disability are 16%–62% less likely to meet physical activity guidelines compared to their peers without a disability (7). These results were further highlighted through the 2022 Canadian Physical Activity Report Card for Children and Adolescents with Disabilities, with overall physical activity graded as a “D” and active play and 24-hour movement behaviours both receiving a grade of “F” (8). As 13% of Canadian children and youth experience functional difficulties in at least one domain (9) and one in five Canadians over the age of 15 identify as having one or more disabilities (10) the need for inclusive and adapted physical activity programming is evident. One method to combat these low levels of participation is by developing physical literacy (5).

Physical literacy describes how confidence and motivation, social participation, movement competence, knowledge, and enjoyment influence engagement in physical activity (11). In 2019, Cairney et al. expanded on this concept further through their holistic framework linking physical literacy, physical activity, and health (5). The model depicts the bi-directional influence between physical literacy and physical activity, while explaining how both can be mediators for physical, mental, and social health outcomes (5). Due to the positive influence physical literacy can have on physical activity levels, physical literacy-based programming is an important intervention method for lifelong physical activity and health promotion.

The Acadia Sensory Motor Instructional Leadership Experience (S.M.I.L.E.) program pairs university student volunteers with individuals with any type of disability three years of age and older (with no upper age limit) from the surrounding community (12). It promotes the development of physical literacy using social, cognitive, and motor goals as a way of tailoring and adapting physical activity to the needs of each individual participant (12). Typically, the program uses high-quality university facilities, equipment, and in-person programming to create a positive environment where all participants are supported and encouraged to be physically active (12). In March 2020, the outbreak of COVID-19 resulted in a global shutdown and provincial public health orders, restricting all interpersonal and hands-on activities for public health and safety (13). In response to this lockdown, many programs, including the Acadia S.M.I.L.E. program, altered the structure of their programing, shifting to an online adapted physical activity program. However, the gap in the literature in this area of study at the time resulted in little direction guiding the creation, implementation, and expected outcomes of an online adapted physical activity program.

In the few pre-COVID examples of online physical activity-based programming, a study conducted by Dyment and colleagues (14), focusing on online physical education programming within the typically developing population concluded that if properly planned, implemented, and supported, online physical activity programming can be effective. At the time of the current study however, there was little research to guide the creation or implementation of online adapted physical activity programming. Thus, the use of virtual programming for individuals with disabilities, in general, was also explored. For example, the use of virtual programming for individuals with disabilities has been shown to have a positive effect on the learning process (15–19). The use of virtual simulation to practice professional skills in individuals with autism spectrum disorder (ASD) was shown to be beneficial as the amount of in-person interaction was limited and supplemented with a less intimidating medium (15). A study conducted by Reicher (19) discussed similar results when addressing the positive potential of a hybrid education for individuals with ASD through the partial removal of the social overstimulation felt during complete in-person education.

More recently, virtual physical activity programming for individuals with disabilities has been investigated. A pilot study conducted by Sharma and colleagues (20) focused on the outcomes of a virtual adapted physical activity program for youth with physical disabilities, with results indicating high program compliance, social cognitive benefits, and increased self-reported physical activity behaviours among participants. The use of online pre-recorded physical activity interventions for adults with intellectual disabilities was also found to be a feasible program option that presents benefits to physical activity participation and overall health for its participants (21).

While the forced virtual shift due to COVID-19 resulted in the Acadia S.M.I.L.E. program altering program delivery, the gap in the literature provided little support in the creation or expected program outcomes. Recent studies provided positive feedback on virtual adapted physical activity programs (20, 21); however, as online adapted programming is a relatively new area of research, the full impact of virtual programming is unknown. Therefore, this program evaluation examined the feasibility and impact of using an online platform to deliver adapted physical activity programming to improve participant physical literacy and physical activity.

## 2. Materials and methods

### 2.1. Design

A mixed-methods case study approach with constructivist roots was used within this study to allow for a comprehensive understanding of program impact on both the participants and their families. The study included a set of questionnaires and interviews completed by the caregiver about their child's participation, in addition to interviews completed by the program leaders, to measure program feasibility and overall program impact over the eight weeks of virtual programming.

### 2.2. Participants

A convenience sample of families were recruited through a study recruitment email sent to participants who had previously been enrolled in S.M.I.L.E. programming. To be considered eligible to participate in this study, an individual must have either had the cognition and English proficiency to read, write, and speak on their behalf (for young adult participants living independently), or had a caregiver with the cognition and English proficiency to read, write, and speak on their behalf. Given that S.M.I.L.E. was developed for individuals with any type of disability three years of age and older (with no upper age limit), there were no age, gender, or diagnosis restrictions to participate in this study.

### 2.3. Virtual S.M.I.L.E. Program

To stay consistent with typical S.M.I.L.E. program delivery, two program groups were created with each group participating in eight weeks of programming. Group one, the Friday night group, consisted of participants aged 13 and older while, group two, the Saturday morning group, generally consisted of participants aged 12 and under with a few older participants as requested by families. The program days and group structure were designed to align with typical in-person S.M.I.L.E. The virtual programming occurred in two methods: (a) asynchronous at-home activity guides; and (b) live virtual Zoom-sessions. The at-home asynchronous activities were identical across both program groups, but the live Zoom sessions were created and implemented independently by the respective leader team. An example of an at-home activity guide can be found in the Supplementary materials.

The at-home activity guides were sent by email at the beginning of the programming week and contained a variety of physical activities that could be completed outdoors or indoors with all members of the family. While the guides contained information on how to complete each activity, there were no instructions on how many activities must be done or the frequency and duration in which the activities should be completed. These guides were sent home on the participants' day of programming, either Friday or Saturday, regardless of whether there was a virtual programming session scheduled for that week.

The live Zoom sessions occurred on three out of the eight weeks of programming. The sessions lasted 45 min in length and were led by the S.M.I.L.E. student leaders; however, unlike the typical in-person S.M.I.L.E. there was no one-on-one pairing with student instructors and participants. These sessions occurred during the third, sixth, and eighth weeks of programming. For the Friday night programming, all participants would join a Zoom call through an email link sent earlier that day. The Friday Night virtual S.M.I.L.E sessions were more social based with much lower amounts of physical activity opportunities. There were 8–10 leaders who met in one Zoom session with the participants (anywhere between 15 and 20) to complete an opening activity together (10 min). The larger group then moved to break out groups for specific activities that met the needs and interests of the specific participants. The participants were organized in groups based on their interest and needs. There were two leaders in a breakout room with 2–4 participants in that section of programming. The activities varied from movement games to cognitive activities and discussions depending on the leaders and the participants (35 min). After the 35 min of programming was completed, participants left the call, while the leaders had a debriefing session as to how the programming session went and to discuss any difficulties that may have arisen.

For the Saturday morning programming, all the participants would join the Zoom call through the link that was emailed in the morning before the program began. The programming session began similarly to the Friday night programming; however, there were no break-out rooms created. The Saturday morning sessions were one larger session as there were less participants interested or available for the virtual setting. There were 5–7 leaders with the same number of participants depending on the day. The session was led as a group session. It was highly interactive, and movement based with all participants engaged in the activities. After the 45-minute call, the participants would hang up, and the leaders, program director, and student directors would have a debrief on the session.

Programming for both Friday and Saturday sessions was planned, created, and implemented by leaders from their respective night. Each group on Friday session programming had different activities planned. The virtual activities were proposed and presented to a supervising S.M.I.L.E. student director earlier in the week before the programming began. An example of a Friday night session and a Saturday morning session can be found in Table 1.

**Table 1:** Example zoom session outline for Friday and Saturday programming.

Program day	Activity	Duration (min.)	Description
Friday Night	Participants Arrive & Group Game	0–10	Participants log onto call, socialize with leaders, do ice breaker activities (freeze dance), Simon says etc.).
	Divide into Break Out Groups	10 (±2)	Participants and leaders join break out rooms to smaller programming groups
	Small Group Activity #1: Activity Chain	10–22(±2)	Participants and leaders take turns creating a movement skill/pattern to perform. The group performs the activity while saying the name of the activity and the person who created it (ex. Marching for Jessica). Each round, a new activity is added to the old activity until a chain of 5–10 activities are being performed (ex. March for Jessica, Hop for Mark, Toe touch for Nate, etc.). Activity increases in difficulty by continuing to add activities or increasing speed.
	Small Group Activity #2: Charades	22–34 (±2)	Participants and leaders take turns choosing an action or thing that match the day's program theme (ex. Decorating Christmas tree for Holiday theme). Leaders and Participants try to guess what the activity the participant is acting out and the person who guesses correctly is the next person to act out the clue.
	Small Group Activity #3: Freeze Dance	34–45 (±2)	Leaders play songs through their computer to all the participants. When music is on, everyone dances, when music stops, everyone freezes.
	End of Program	45 (±2)	Leaders wrap up final activity of the night and say goodbye to all participants. Once all participants exit break out group and log off, leaders join program debrief session.
Saturday Morning	Participants Arrive	0–5	Participants log onto call, socialize with leaders
	Activity #1: This or That	5–15 (±2)	Leaders use “Present Feature” to show screen graphic. Two options show up and leaders ask which option each participant would rather do/have (ex. Hot chocolate or Tea). Each option has an action attached; thus, participants have to perform the action to make their choice (ex. Choose Hot Chocolate means “Stretch Arms to the Sky” and Tea means “Stretch Arms to the Floor”). Once everyone makes their choice, another set of options appear.
	Activity #2: Active Story Time	15–25 (±2)	One leader tells a story associated with the theme of week (Holiday theme: Story about going on a Ski trip). The leader moves and acts out the activities in the story as it is told and prompts all participants to follow along (ex. “Then we walked to the hill. Everyone March with me!” Then we put on our gear. Everyone grab your boots and put them on (mimics putting on big boots)”.
	Activity #3: Household Scavenger Hunt	25–35 (±2)	Leaders take turns choosing a category of items commonly found in houses that fit the theme of the week (ex. Holiday theme: Grab hats). Leaders then put on a timer song and each participant leaves their screen and rushes to find the item in their house and get back to the screen before the timer is done. Then all participants present their objects. After presenting their objects, another category of object is given and the game repeats.
	Activity # 4: Freeze Dance	35–45 (±2)	Leaders play songs through their computer to all of the participants. When music is on, everyone dances, when music stops, everyone freezes.
	End of Program	45 (±2)	Leaders wrap up final activity of the night and say goodbye to all participants. Once all participants exit break out group and log off, leaders join program debrief session.

### 2.4. Procedure

Data collection occurred in four stages: (a) pre-survey; (b) weekly check-in surveys; (c) post-survey; and d) post-programming interviews. All measures were completed by caregivers, except for the instance where an adult participant lived independently and completed the measures themself. For anonymity and clarity, regardless of whether a caregiver or an adult participant completed the measures, the participant data will be referred to consistently as collected from caregivers. Post-programming interviews were also conducted by S.M.I.L.E. program leaders from both Friday night and Saturday morning. Questions for the post-programming interviews differed between caregiver and leader interviews to ensure all aspects to program creation, implementation, and participation were adequately captured.

#### 2.4.1. Pre- and post-survey

Caregivers completed a brief online survey pre- and post-intervention. The survey contained questions regarding each participant's demographic and disability information, physical literacy, and physical activity.

Physical functioning was assessed with the Washington Group Short Set of Questions on Disability, a valid and reliable measure of functional difficulties (22). Across the five categories: vision, hearing, mobility, cognition, and self-care, the caregivers rated their child on a scale from 0 to 4 with 0 being “no difficulty” and 4 being “unable to do” (22). A functional disability rating was calculated for each participant between 0 and 1 with 0 having no difficulties and 1 having severe difficulty (22). These questions were only included in the pre-survey and the internal consistency was acceptable (Cronbach's alpha = 0.61).

Emotional functioning was assessed using a subset of questions from the Pediatric Quality of Life Inventory, a valid and reliable measure of health-related quality of life (23). Caregivers reflected on their child's frequency in which they feel specific emotions, (scared, sad, angry etc.,) (23). These were rated on a 5-point scale, with 100 indicating that there is “never” a problem and 0 indicating that there is “almost always” a problem (23). These questions were only included in the pre-survey and the internal consistency was good (Cronbach's alpha = 0.84).

The PLAYparent was used to assess the cognitive (confidence, motivation, and comprehension), motor (locomotor and object control skills), and environmental (participation in water, indoor, outdoor, and snow/ice activities) domains of physical literacy (24). The survey consisted of 19 questions each measured on a 3-point scale, with 0 indicating low and 2 indicating high. Responses were averaged to provide domain and total physical literacy scores. The PLAYparent has previously demonstrated good construct and convergent validity (25), and the internal consistency in this study was good both pre- (Cronbach's alpha = 0.91) and post-intervention (Cronbach's alpha = 0.92).

The International Physical Activity Questionnaire for Adolescents (IPAQ-A), a valid and reliable measure of physical activity, was used to assess the number of minutes in a week the participant engages in leisure-time physical activity (LTPA) (26). LTPA encompasses all physical activity that is performed by a person outside of the requirements of daily tasks, such as leisure activities, recreational activities, organized sport participation, and exercise or workouts. Light, moderate to vigorous, and total LTPA scores, in minutes per week, were calculated.

#### 2.4.2. Weekly check-in survey

At the end of each of the eight weeks of programming, data were collected through a two-to-three-minute online survey. After each week of programming, a link to the weekly checklist was emailed to the caregiver of the participants specifically on their programming day. For example, Friday participants would receive a link to a survey on a Friday to reflect on the previous week of programming, regardless of whether there was a Zoom session. The survey contained questions on the number of days in the week the participant met physical activity guidelines (out of seven), as well as if the at-home activities for the week were completed and if so, how often they were completed (out of seven), and the overall enjoyment and ease of completing the activity (each out of ten). If the at-home activity was not completed, participants could indicate a reason they were not completed.

#### 2.4.3. Post-programming interviews

##### 2.4.3.1. Student leader interviews

After the 8-week programming, four leaders were selected to be interviewed on their experiences, and opinions about the program. The leaders, two from Friday programming, and two from Saturday programming were purposively selected by availability. The interviews were conducted through a video-chat program and ranged from 9 to 17 min in length. The researcher conducting the interviews was a student-researcher affiliated with the 8-weeks of programming; however, all four leaders were briefed that any comments made would be held confidential, would not be shared with any program director with their name affiliated to the comment, and would in no way affect their current or future role in the S.M.I.L.E. program. Within the interview, the leaders were asked questions about their perception on the structure, feasibility, benefits, strengths, and weaknesses of the program. The semi-structured interview (see Supplementary material) provided each leader with the opportunity to include anything they found relevant about the program while also speaking to specific topics as guided by the researcher.

##### 2.4.3.2. Caregiver/participant interviews

The interviews were completed through a video-chat program and ranged from 8 to 25 min in length. The researcher that conducted the interviews was not previously involved with the Acadia S.M.I.L.E. program. Within the interview, the caregivers and the participant were asked questions addressing the impact, feasibility, and overall impressions of the program for families. The semi-structured interview (see Supplementary Material) provided the interviewee with the ability to speak freely about their thoughts and feelings surrounding the program.

#### 2.4.4. Data analysis

Missing data on the PLAYparent was dealt with through mean imputation to ensure that the motor, cognitive, environmental, and total physical literacy scores could be calculated. There were five instances of missing data where mean imputation was used.

Descriptive statistics (mean, frequency, etc.) were used to describe the sample in terms of demographics and disability-specific information. Descriptive statistics were also employed for the weekly surveys to describe rates of participation and enjoyment. Paired samples t-tests with an alpha value of 0.05 were performed to assess the impact of the program on physical literacy and physical activity from pre- to post-program. Effect sizes were reported using Cohen's *d* with 0.2, 0.5, and 0.8 indicating small, medium, and large effects, respectively (27). All quantitative analyses were performed in Jamovi software version 2.0.0.0.

Caregiver and leader interviews were recorded into audio files and then transcribed using the Microsoft Transcribe feature in the online Microsoft Word program. These transcriptions were then edited to ensure consistency with the audio file and reviewed numerous times to develop a thorough understanding of the experiences that were expressed by the participants. Reflexive thematic analysis was used to identify and analyze the themes in the data. The six phases of Braun and Clarke's (28) guidelines to reflexive thematic analysis were implemented. The six phases included: (a) familiarization with the dataset; (b) developing initial codes; (c) constructing themes; (d) reviewing potential themes; (e) defining and naming themes; and (f) writing the report. Reflexive thematic analysis provides flexibility in its application to a range of theories as well as provides flexibility for the researcher's decision on themes and types of analysis (29).

## 3. Results

The study was completed by 16 S.M.I.L.E. participants between Friday night and Saturday morning programming days. After the pre-surveys were completed, one participant did not finish any other survey or questionnaire, leaving 15 participants with complete data for analysis. Participants ranged between 7 and 31 years of age with six participants being children/youth (under 18 years of age) and nine participants being adults (over 18 years of age). Eleven caregivers responded on behalf of their child/children for data collection. Two of the caregivers had two children enrolled in the program, thus they responded for both participants; one caregiver responded on behalf of three participants; and one participant who lived independently responded on their own behalf. Complete demographic information is presented in Table 2.

**Table 2:** Participant demographic information.

Variable	Mean (SD) or Frequency (%)
Age (years)	20.4 (6.87)
Sex	
Male	7 (47%)
Female	8 (53%)
Diagnosis	
Autism Spectrum Disorder	9 (60%)
Down syndrome	3 (20%)
Intellectual Disability	1 (6.6%)
Global Developmental Delay	1 (6.6%)
3rd Chromosome Depletion	1 (6.6%)
Physical Functioning	0.13 (0.12)
Emotional Functioning	53 (22.5)

### 3.1. Survey data

Change in physical literacy and physical activity from pre- to post-intervention is presented in Table 3. The PLAY cognitive score had a statistically significant change with a decline of 0.13 between the pre-survey and post-survey (*p *= 0.03). It was the only variable to have a medium effect size (*d *= 0.63). All other measures showed no significant change between pre-and post- surveys and all effect sizes were small. When looking at physical activity, light, moderate-to-vigorous, and total physical activity all increased over time; however, these changes were not significant, and the effect sizes were small.

**Table 3:** Change in physical literacy and physical activity from pre- to post-intervention.

Domain	Variable	Pre mean (SD)	Post mean (SD)	*p*-value	Effect size (Cohen's d)
Physical Literacy	Cognitive	1.04 (0.57)	0.91 (0.53)	0.03	0.63
	Motor	1.17 (0.36)	1.13 (0.50)	0.74	0.09
	Environment	0.92 (0.39)	0.93 (0.62)	0.91	0.03
	Total	1.08 (0.39)	1.02 (0.47)	0.53	0.17
Physical Activity	Light	92.33 (91.34)	102.67 (140.74)	0.67	0.11
	Moderate to Vigorous	66.33 (74.41)	75 (113.12)	0.68	0.11
	Total	158.67 (142.4)	177.67 (243.24)	0.64	0.13

The weekly survey had an average response rate of 81.25%, with the highest response rate on week three at 93.75%, and the lowest response rate on week eight at 62.50%. Of the weekly responses collected, the data indicated low at-home activity completion (as indicated by participants saying they performed the S.M.I.L.E. activities at least one time that week). The week with the highest completion of the programming was week eight closely followed by week four at 60.00% and 58.30%, respectively. Week three had the lowest completion rate with 20.00%. Average completion throughout the eight weeks of programming was 42.30% (SD = 13.44). Although week eight had the highest activity completion rate, it also had the lowest survey response completion. Inversely, though week three had the highest survey response completion, it had the lowest activity completion rate. The most common rationale for not completing programming was that “My child wasn’t interested” and “We didn’t have time” with these responses being used 19 and 16 times, respectively, over the eight weeks of programming.

The average enjoyment throughout the program, as seen in Table 4, was 8.67 (SD = 1.30) on a 10-point scale. The average ease of completion of the activities throughout the programming was 5.31 on a 10-point scale (SD = 3.11). The perceived ease rating throughout the eight weeks had the highest SD showing large variability within the measure. Participants reported that the 60 min per day physical activity guideline was met, on average, 3.27 days (SD = 1.53) per week.

**Table 4:** Descriptive data from weekly survey responses.

Measure	*N*	Mean	SD	Min	Max
Weeks of Programming Completed	15	2.87	2.88	0	8.00
Program Enjoyment	11	8.67	1.30	6.67	10.00
Perceived Ease of Activities	11	5.31	3.11	1.00	9.50
Days Active with S.M.I.L.E. Activities	11	2.24	1.22	1.00	4.71
Days 60 min MVPA Achieved	15	3.27	1.53	1.29	6.25

If a participant responded that they did not complete programming that week, they were not prompted to report on the enjoyment, ease of activities, or days active with activities for that respective week.

### 3.2. Interview data

Post-programming interviews were conducted with six caregivers on behalf of eight participants with two caregivers speaking on behalf of their two children. One interview was completed by an adult participant themself. For anonymity and clarity, all quotes from caregiver interviews will be sourced as a caregiver despite one interview being completed by a participant directly. Of the four leader interviews, two were conducted with leaders from the Friday night program, and two from the Saturday morning program.

From the total of eleven interviews conducted, five main themes arose in relation to program participation: (a) program impact on physical activity; (b) virtual impact on programming; (c) program impact on social and motor goals; (d) impact of program design; and (e) program feasibility for families. These themes, subthemes, and related quotes are presented in Table 5.

**Table 5:** Themes and subthemes from the caregiver and leader interviews.

Theme	Sub-theme	Example quote
Virtual Impact on Programming	Few Perceived Virtual Barriers for Participants	"For us because we had already been doing it for quite some time, I was used to it from school because I was on an I was doing stuff like meetings through zoom for a while and uhm we also do speech therapy on a different platform and so we’re kind of used to all that stuff. But in the beginning of covid, if you had started it, I probably wouldn’t be quite so tech savvy.” [Caregiver 1]
	Virtual Barriers Posed Slight Problem	“We had a couple of Wi-Fi issues. Sometimes people had just difficulties like opening zoom links when we’ve changed the links, but once they got the second hand on it, overall, it was pretty straightforward for most families. I know that some families don’t have like reliable access to Internet, so that sometimes is struggle for them.” [Leader 4]
	Virtual Programming Posed Problems for Activity Transitioning	“I do find for [Participant's Name] I should never tell her [Virtual S.M.I.L.E. is] happening until it’s actually up and running. So, waiting to log in so waiting to be invited into the sessions with just the blank screen when she thought it was going to be S.M.I.L.E. […] If there’s those hiccups and it’s not right there when she’s anticipating it, that can throw her off as well.” [Caregiver 8]
	Enjoyed/appreciated use of Technological Tools (Email, Zoom Features etc.)	[Discussing enjoyment of the Spotlight feature on Zoom] “Being able to spotlight [the participants] on the screen really gave them the chance to speak instead of like at regular S.M.I.L.E. when you wouldn’t necessarily have that.” [Leader 3]
	Virtual Platform Helped Reduce some Anxiety for Participants with ASD	“[In in-person S.M.I.L.E.] “[They were] super shy, low engagement, you know high needs ASD, but [they] shone like [they were] great with the online stuff. I think it was just like [they were] kind of less kind of, [they were] less distracted you know there was less kind of external stimuli going on.” [Leader 3]
Program Impact on Social and Motor Goals	Perceived Social Benefit	“If you could only have seen how happy she was when she was in those zoom classes and she was seeing familiar faces. Not only of the leaders but they you know they had other people in there as well that she knew she was just ecstatic just for that, that connection itself”. [Caregiver 4]
	Difficulty Motor Programming	“You can’t really set up a relay race like something like that, or like an obstacle course, something like that you do at S.M.I.L.E, you could, we could normally do, but it did open up some other like opportunities where we got to play a little bit with the technology. […] So it did kind of like open up that opportunity but you know it did close some doors as well.” [Leader 3].
Programming Impact on Physical Activity	Physical Activity Levels Decreased	“I would have to say she was less active, my daughter before covid she was busy four days a week after school, for various activities, at least four days a week, and [um] with covid that went down to zero at one point and then getting her to connect with the S.M.I.L.E. programming to do things to be physical was difficult.” [Caregiver 4]
	Physical Activity Levels Constant or Increased	[Discussing impact of programming on physical activity and levels may have increased but] “not really that much more”. [Caregiver 8]
	Program Influenced Family Physical Activity Levels	“In the last while we have not been very good at that, like just even as caregivers, we haven’t been having much physical activity in the last while, so it actually encouraged us. I’m doing 30 min a day now.” [Caregiver 7]
Impact of Program Design	Liked the At-Home Activities	“She loved the at-home activities” […] “it was great for me as a tool as well to have a resource to go to for ideas to do something different” [Caregiver 6].
	At-Home Activities Not Well Received	“I always read the activities to her and I think the first week we managed to do a couple of them, but after that it was totally downhill.” [Caregiver 4]
	Zoom Sessions Were Well Received and/or Should be More Often	“I’ll tell you the three zoom classes that were held. She totally loved those and she participated in the activities and it- by the time we got through to the third one I wasn’t even in her room with her.” [Caregiver 4]
	Participants/Caregivers Missed 1-to-1 Pairing as in In-Person Programming	“And you know the special relationship that they get with the partner they have in S.M.I.L.E. like there’s nothing like it. There’s literally nothing like it so that I knew would be kind of a sad thing because she didn’t have like one person that kind of just dote on her, which you know, something that she really looked forward to.” [Caregiver 11]
Program Feasibility for Families	Liked the use of Outdoor Involvement in Activities	“It gave us things we could do within our own little neighbourhood within our own yard that still kept her busy and engaged in having fun and it didn’t necessarily involve a TV or computer or again all those everyday things she has.” [Caregiver 6]
	Location Problems with Programming At-Home	“My place isn’t really set up for that.” [Caregiver 8]
	Time Requirements Were Difficult	“That’s where the problem came in and that’s why we didn't get more of [the At-Home Activities] done is I don't have, I didn't have the time like between my husband and I we both work different shift work, so we basically try to work like opposite schedules. So we're always crazy.” [Caregiver 11]
	Program reduced Location Barriers	“We've relocated. It was just a relief to know that there was still going to be a connection of some sort with the program” [Caregiver 6]

## 4. Discussion

The purpose of this study was to examine the impact of a pilot virtual adapted physical activity program on the physical literacy and physical activity of individuals with disabilities; along with assessing the feasibility of the program. When comparing pre- and post-program physical activity and physical literacy scores, results showed that the cognitive physical literacy domain, which measures confidence, motivation, and comprehension, showed a statistically significant decrease, while no other measure showed significant change. As shown through other studies on physical activity and physical literacy levels during the COVID-19 lockdown, child physical activity levels (30–32), adult physical activity levels (33), and caregiver reported child physical literacy (30), decreased as a result of the COVID-19 pandemic. While the downward trend in cognitive physical literacy data from this study is consistent with the general decline in physical literacy in one study (30); the lack of a statically significant decline in all other physical literacy domains, as well as all physical activity measures in the current study are optimistic and suggest that the program helped participants to maintain their physical literacy and physical activity over the eight weeks.

Using the Online Physical Education (OLPE) framework (34) as a lens to review the virtual S.M.I.L.E. program, key moderators within the OLPE for program success such as technology access, community factors, programming components, and family factors were identified to assess program efficacy and provide insight into the lack of improvement in physical literacy and physical activity for participants. While our interview data suggests that technology issues may have been present for some families, they did not create any significant problems in program access or implementation for participants; however, future investigation should be conducted to determine if program participation was affected due to technological barriers. The community factors for virtual S.M.I.L.E. programming may have also played a role in its effectiveness, as the program switched from having university facility and equipment access to occurring remotely with household items; however, this was not heavily discussed within the interviews thus, future investigation should occur to better understand the impact this had for participants. Through interview and survey data, three common themes arise from the programming and family OLPE framework: (a) resource quality; (b) parental time expectations; and (c) professional training (34). These factors may have contributed to the lack of improvements in physical literacy or physical activity in the current study.

The quality of the at-home activity guides had some highlighted challenges, with activities being called too juvenile or not of interest to participants, indicating that the activities were not adequately created to suit the demographics of the program. When creating the program materials, activities were designed with the intention of catering to a younger demographic, but after program recruitment was complete, the average age of the participants (20.4 years) was much higher than expected, thus explaining the activity-age discrepancy. Caregiver interviews also highlighted that the increased time expectations for the program were difficult to manage. Webster and colleagues (34), identified this problem within their OLPE, discussing that virtual programming can result in a disruption to typical lifestyle habits due to the increased responsibility placed upon the caregivers to aid in program implementation (34). As the primary two reasons for not completing the at-home activities, as identified through the weekly check-in surveys, were that “My child wasn’t interested” and “We didn’t have time”, both the quality of the at-home activity guides and the additional time requirements from parents were identified as barriers and a large contributor to low at-home activity program adherence.

Additionally, the leader interviews identified that motor skill programming, a large component of in-person S.M.I.L.E., was difficult in the virtual environment. Leaders indicated that they struggled with motor programming due to the shift in programming options, lack of interest in activities by participants, and reduced equipment and space. While all program leaders were trained to facilitate S.M.I.L.E. programming, online training was much less extensive than in-person, which may explain the discrepancy in the leaders' ability or confidence to program the activities. To improve the quality of motor programming and physical activity engagement for online education, educators and leaders must put additional focus on creating and designing the material to deliver to students (35); thus, additional online physical activity program training should be implemented to help facilitate this creation.

When combining the low at-home activity guide adherence with the fact that the Zoom session activities focused more heavily on social, rather than physical, engagement it is possible that there was simply not enough motor skill and physical activity engagement for participants to improve their physical literacy and physical activity levels. While a recent study showed that online adapted programming was beneficial for physical activity promotion, there was also high program adherence by participants (20). The low program adherence seen in this study could have been a moderator in program efficacy. Additionally, as gross motor programming provides the most benefit after 16 or more hours of intervention (36), with the virtual S.M.I.L.E. program holding three, 45-minute live sessions, coupled with eight weeks of at-home activities, which had low adherence, there was inadequate motor engagement, which could have caused the lack of significant increases in physical literacy or physical activity. Future alterations to the program could include increasing program length and, once again, spending more time and effort to program specifically for gross motor function for maximum benefit.

Additional findings from the caregiver and leader interviews identified that the largest program benefits were in the social domain. With the COVID-19 lockdown preventing pre-existing in-person programming, children and adults alike were shut off from a large source of socialization. As social participation is a component of physical literacy and has a bi-directional relationship with physical activity (5), the benefits of virtual S.M.I.L.E. on the social domain may have had benefits beyond the immediate study impact. However, it is important to note that these results were found at a time when there was a higher number of barriers faced for socialization among participants than usual, thus social improvements may also be partially due to the lack of socialization many participants were feeling in response to the pandemic.

Finally, the virtual S.M.I.L.E. program was found to have specific benefits for a few participants with ASD due to the less stimulating virtual program environment. Leaders three and four expressed that due to the barrier of the screen, less stimulating environment, and less overwhelming programming, participants who typically remained quiet and passive during in-person programming, exceeded previous participation expectations in the virtual programming. These results are consistent with previous literature which focused on a cohort of students with ASD who participated in online non-physical education-based schooling (19). Future research should be conducted to further understand the effects of virtual physical activity programming specifically for participants with ASD.

### 4.1. Limitations

Due to COVID-19, in-person assessments of physical literacy were not possible resulting in all data being collected through the perspective of the caregiver. Miscommunication or different interpretations in the understanding of questions in the surveys may have affected the validity of the responses. Furthermore, all assessments of motor improvements and physical activity levels were not able to be measured and assessed by researchers through objective measures, which may have reduced the overall accuracy of the program's impact on the motor domain of physical literacy and physical activity. Additionally, the age range of participants within the study was large, yet overall, the sample size was small, preventing researchers from conducting age-specific analyses to better understand program effectiveness. Finally, as all assessment tools, check-ins, and activity delivery were online, the technological literacy of the caregivers and/or participants may have impacted participant numbers and overall program participation.

### 4.2. Conclusion

At the beginning of the COVID-19 pandemic, there was a high need for a shift from in-person to virtual programming; however, there was little research to help guide the program creation and implementation. After shifting an adapted physical activity program to a virtual platform, physical literacy and physical activity did not show any significant increases. Throughout the pandemic, there was a downward trend in physical literacy and physical activity behaviours (30–33); thus, the lack of parallel declines for participants in the current study could be perceived as promising for the program. However, due to the low at-home activity adherence and challenges with motor programming in the limited live Zoom-sessions, the lack of a significant increase in physical literacy and physical activity could indicate a need for program adaptations to facilitate improvements. Future applications of virtual programming could see sport and recreational organizations and community centres using a virtual option to supplement their in-person sessions, continue programming when it would have otherwise been cancelled due to inclement weather or facility scheduling complications, or to increase the reach of their programming to rural or hard to reach groups. Future research should be conducted to further understand the impact of virtual physical literacy and physical activity programming for individuals with disabilities, thus allowing a better understanding of external influences potentially affecting program development. Future research should also be conducted to help improve accessibility and better understand barriers for families that typically participate in in-person adapted physical activity programming, but who are hesitant to sign up for virtual programming. Finally, time and research should also be allotted to creating a holistic training program for leaders of online adapted physical activity programming, as well as how to create engaging and age-appropriate programming for all participants.

## Data Availability

The raw data supporting the conclusions of this article will be made available by the authors, without undue reservation.
