# Supplementary material for: Program evaluation of a virtual physical activity program for individuals with disabilities
Source: Front Sports Act Living. 2023 May 2;5:1128565. doi: 10.3389/fspor.2023.1128565 (PMC10185781; doi:10.3389/fspor.2023.1128565)
Supplement: Supplementary file 1 [file Datasheet1.docx]

Supplementary Material

A pilot study of virtual physical activity programming for individuals with disabilities

Nikki Matthews^1^, Roxanne Seaman^1^, Emily Bremer^1*^

^1^School of Kinesiology, Acadia University, Wolfville, NS, Canada

*** Correspondence:**Emily Bremer
emily.bremer@acadiau.ca

# Supplementary Data

1. Example At-Home Activity Guide
2. Leader Interview Guide
3. Caregiver Interview Guide


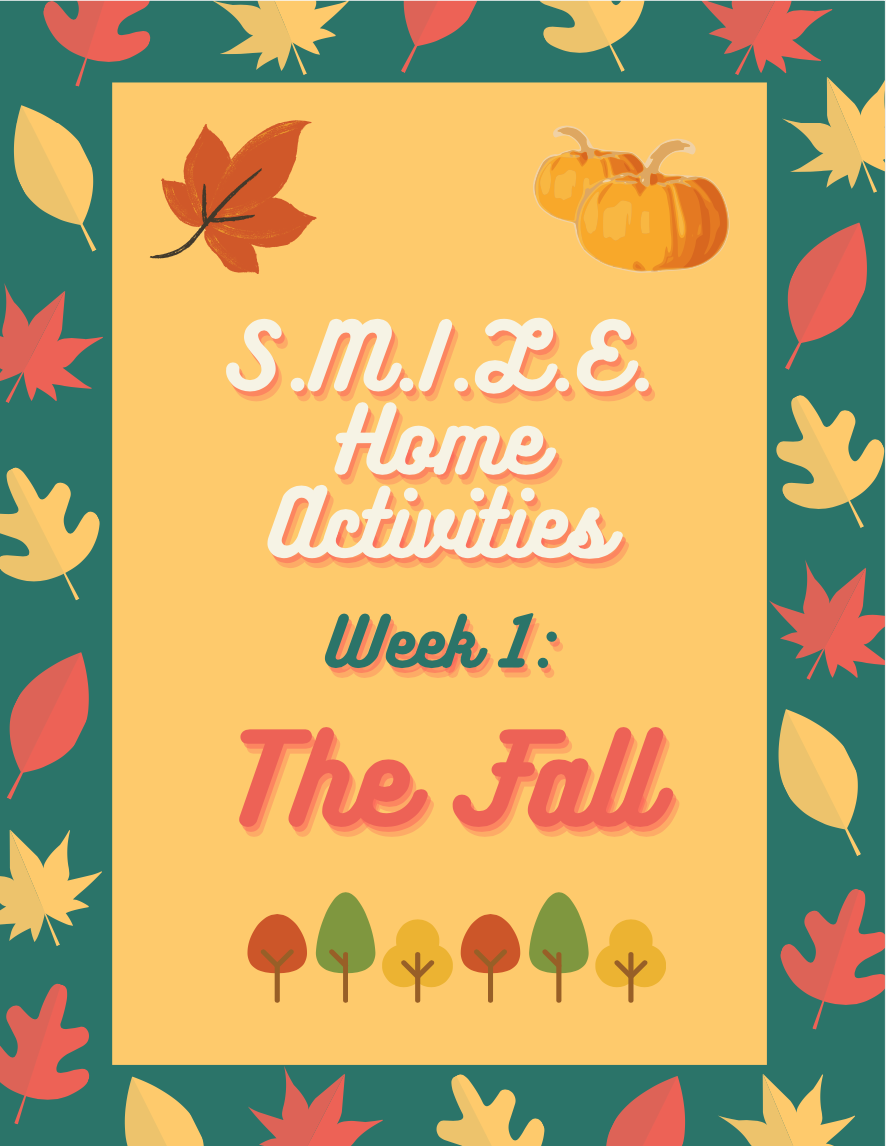


1. Example At-Home Activity Guide


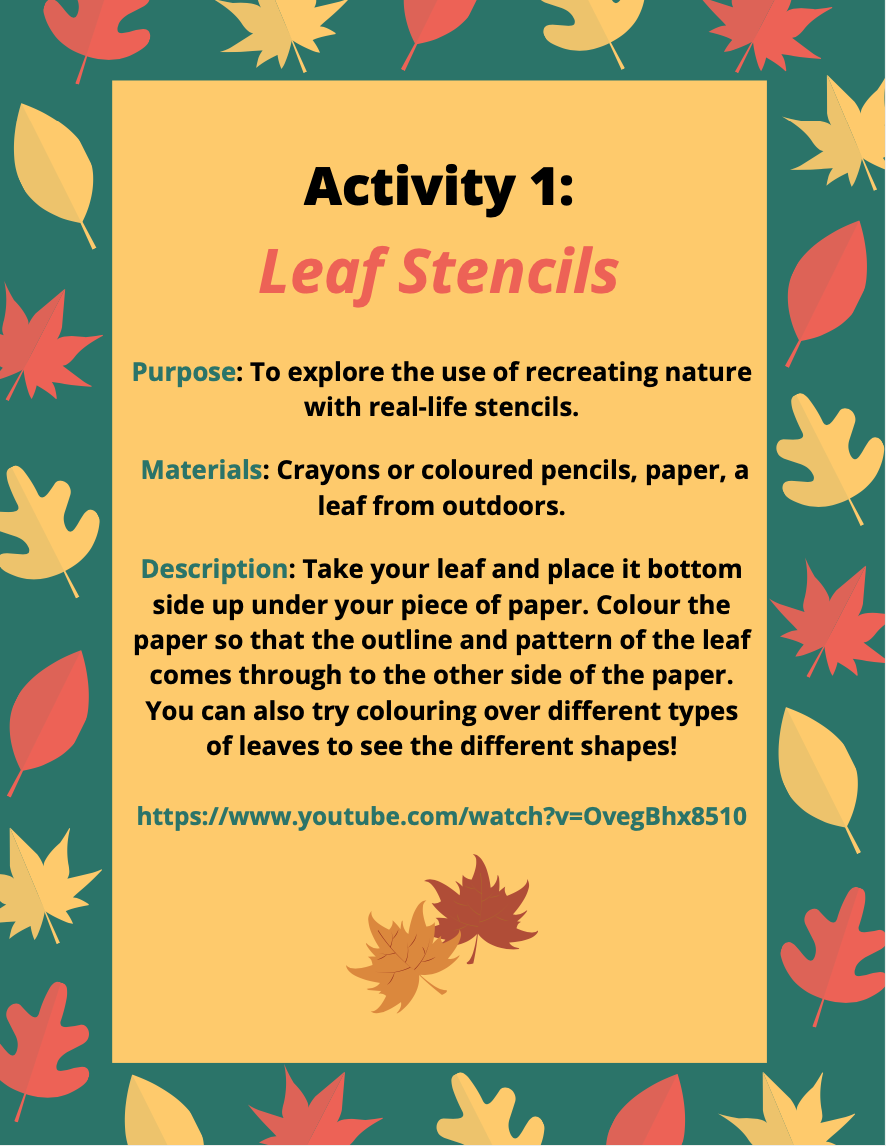

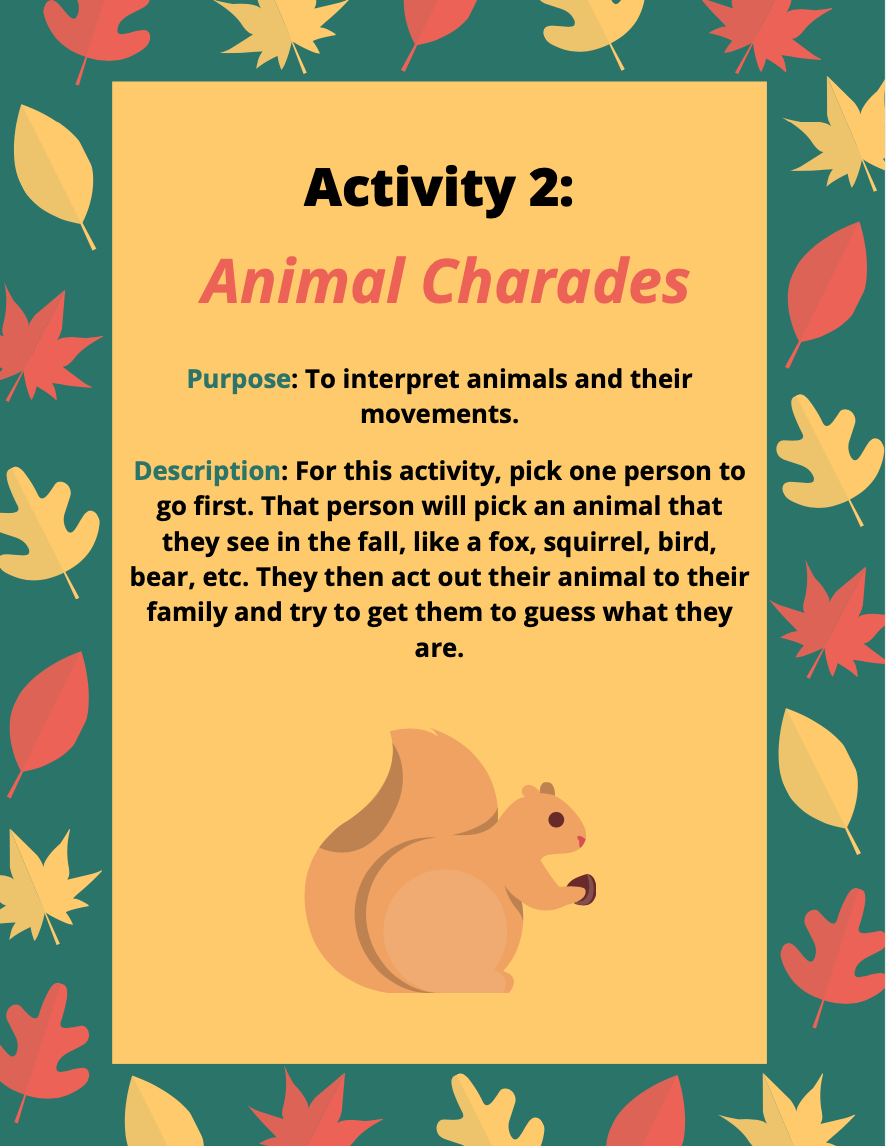

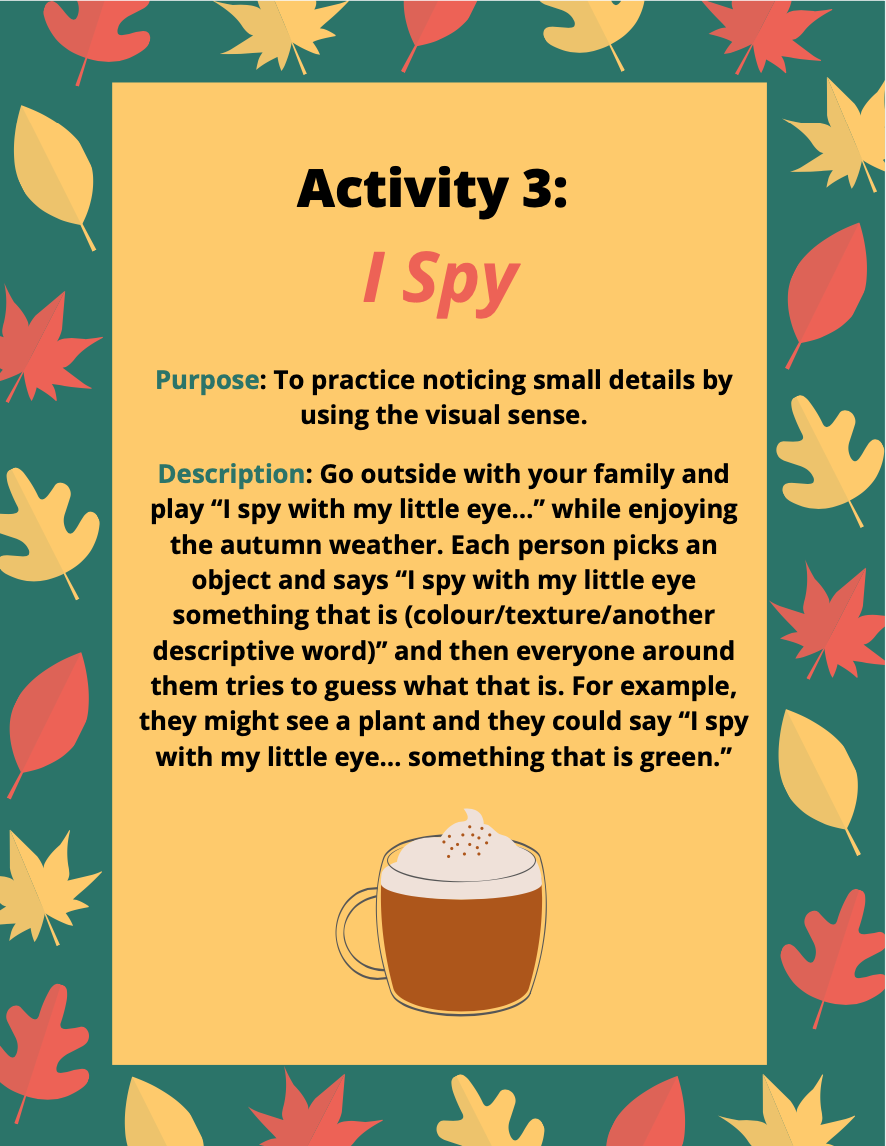

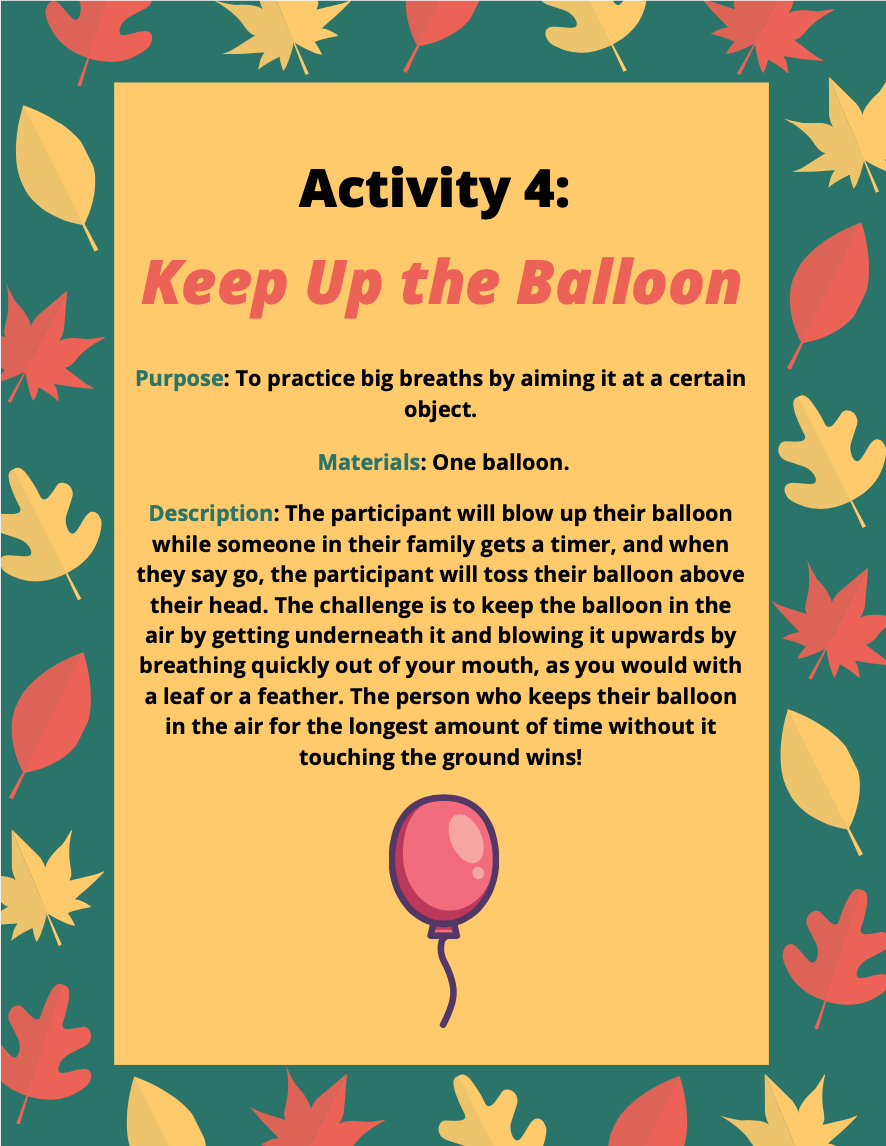

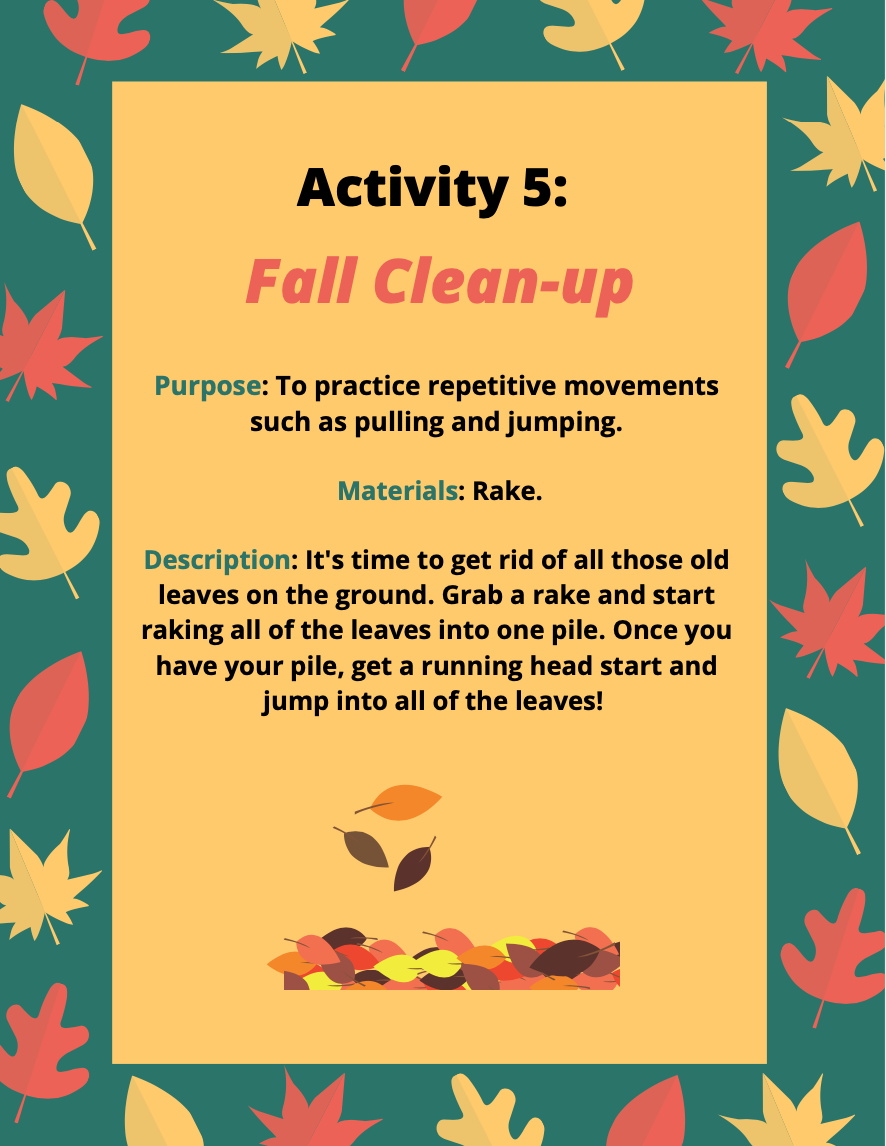

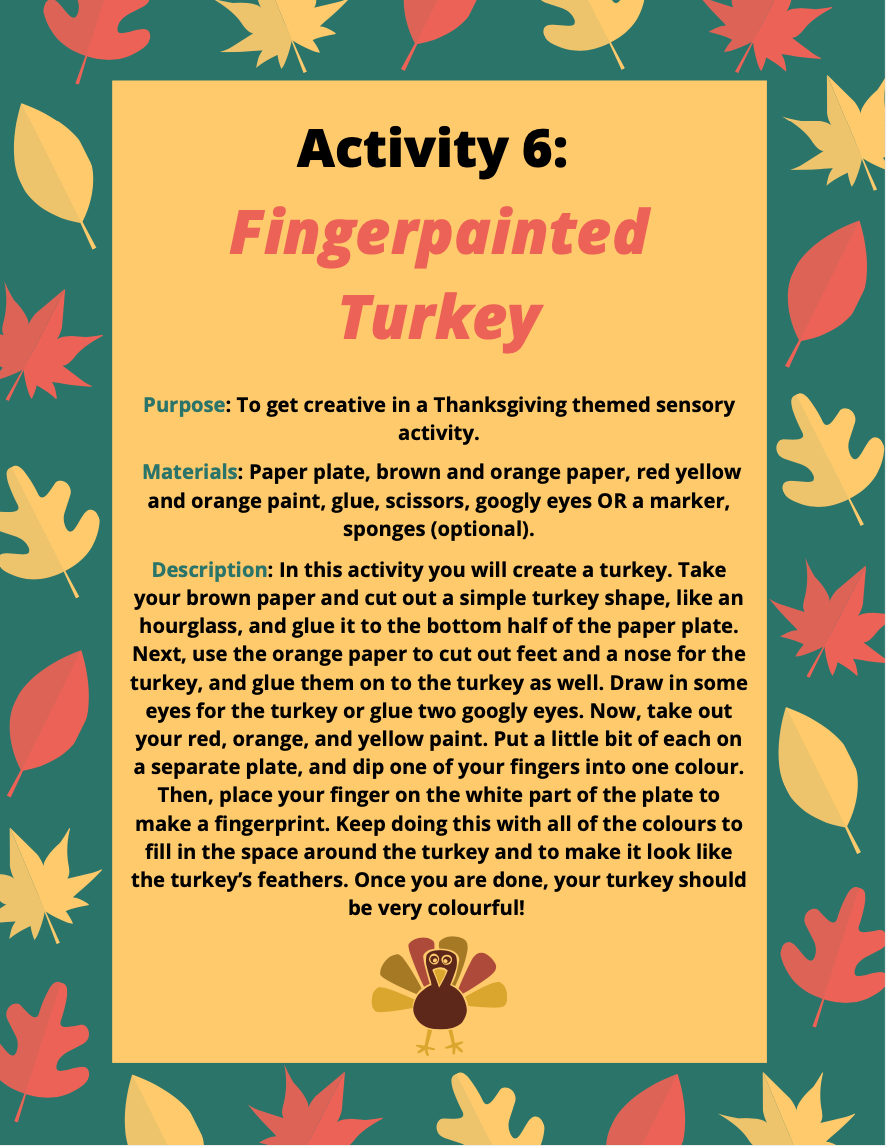

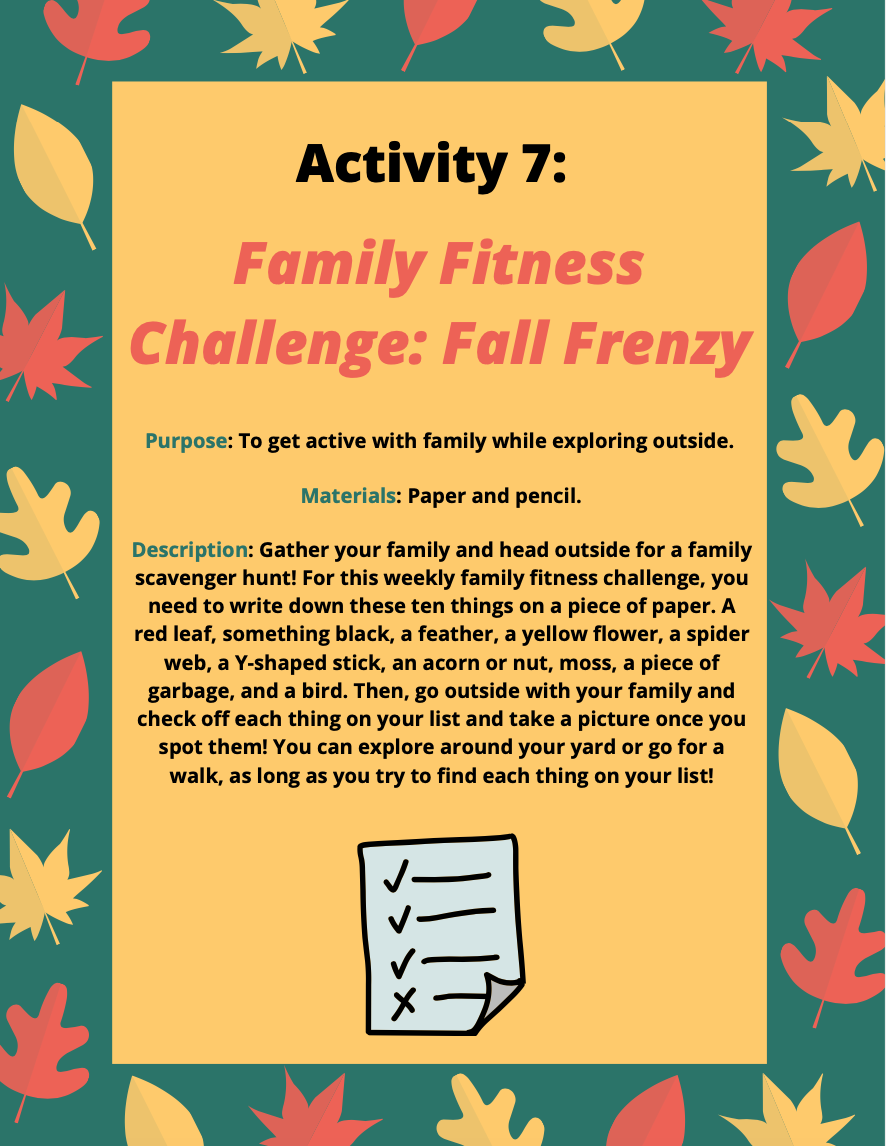

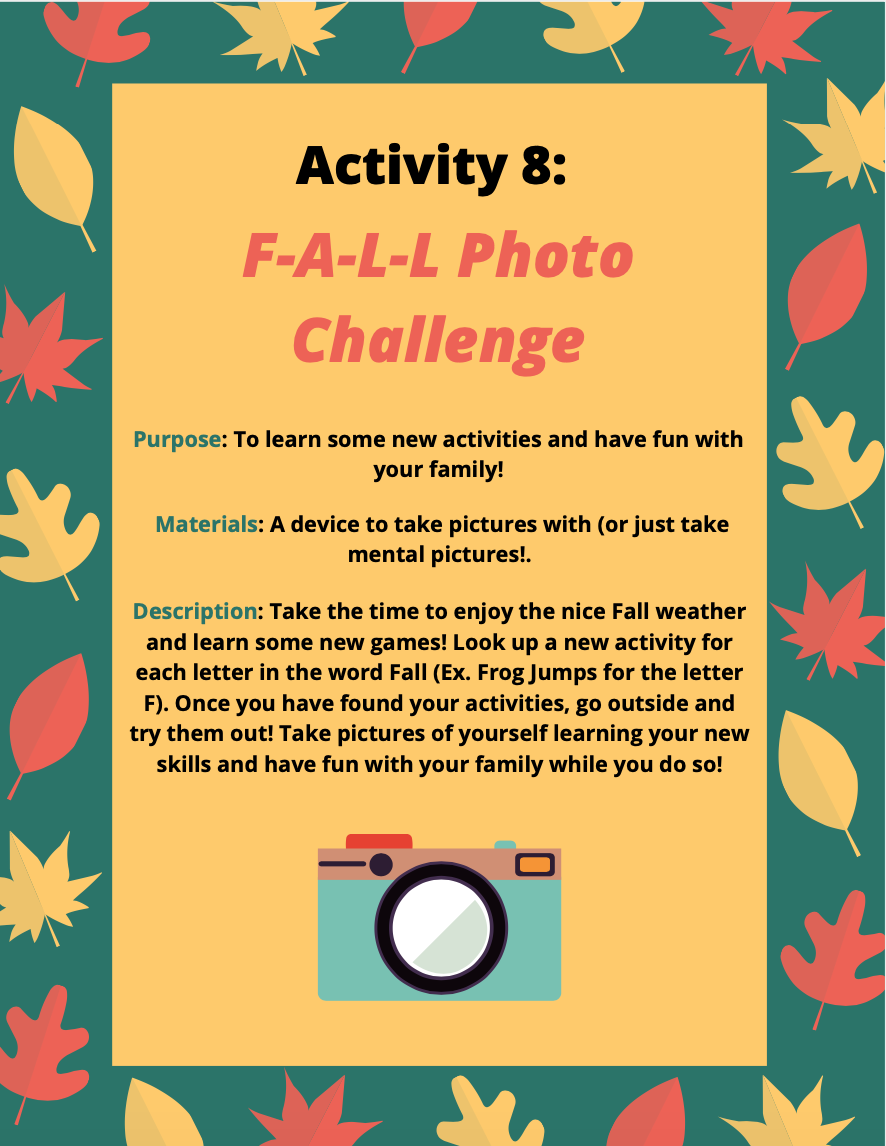


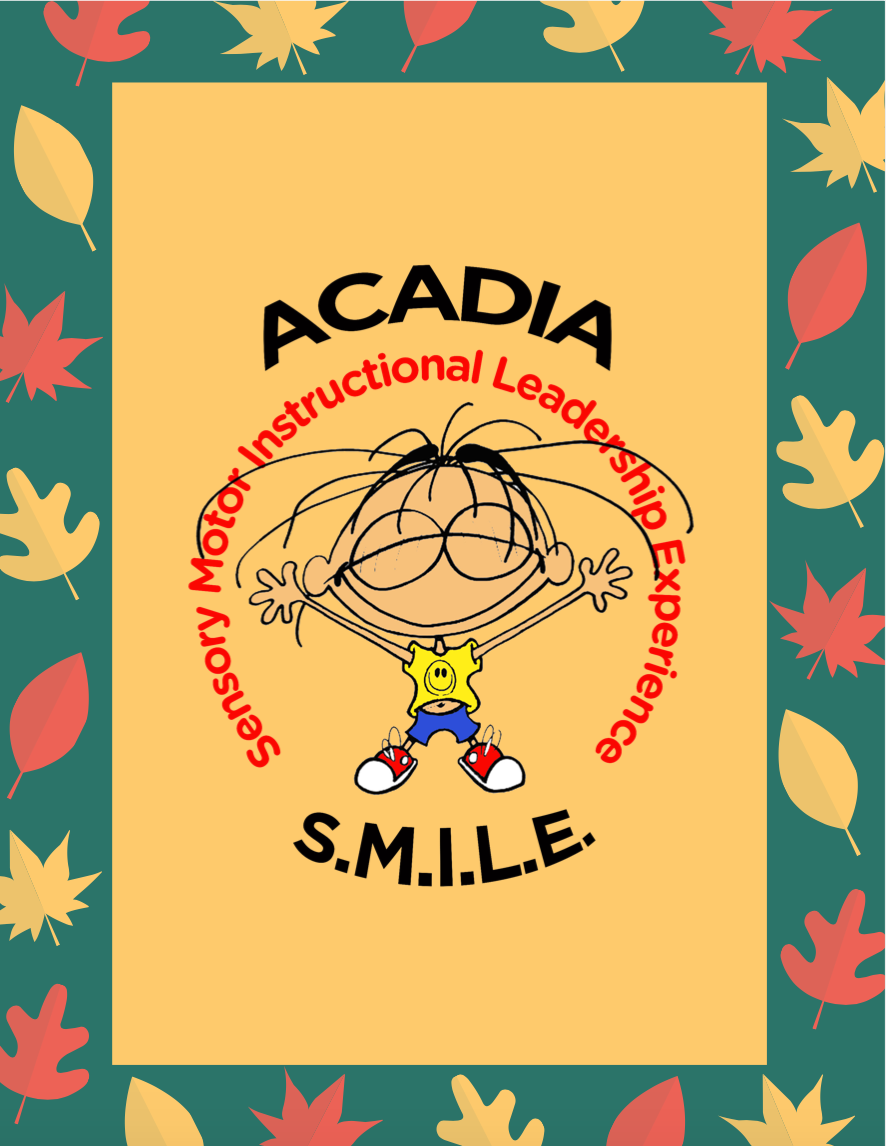


**B. Leader Interview Guide**

The below will act as guiding questions, with room for open-ended responses and follow-up questions:

1. What was your initial impression of changing S.M.I.L.E. from in-person to online programming?
2. How did your programming strategies change when accommodating for online participation?
   1. Was it easier/more difficult?
   2. What were any challenges you faced when planning your activities?
   3. Were there certain activities you used more frequently than others?
3. How did you feel about virtual S.M.I.L.E.?
   1. Did you like the structure of the program
      1. Length (Time)
      2. Number of leaders for each participant
      3. Amount of control over your group (Activities, programming)
   2. Did you have any issues accessing the program? Technology, wifi, etc.?
   3. How did you feel like your group responded to the online activities compared to previous

years of in-person activities?

1. How did online programming meet or not meet your expectations?
   1. Do you think you were able to effectively target motor, social, and cognitive skills through

online activities?

1. What are aspects of the online programming that you liked?
2. What are aspects of the online programing that you did not like?
3. If S.M.I.L.E. was to be moved online in the fall, would you still be interested in participating?
   1. If so, what are any changes you would make to the program?
   2. If not, what are some of the reasons that impacted your decision?
4. Is there anything else you want to tell us about your experience with the instructional aspect or

leadership role in virtual S.M.I.L.E.?

1. **Caregiver Interview Guide**

The below will act as guiding questions, with room for open-ended responses and follow-up questions:

1. What was your first impression of virtual S.M.I.L.E.?
2. How do you think your child felt about virtual S.M.I.L.E.?
   1. Did they enjoy it?
   2. Did they want to practice the activities at home?
   3. Did they talk about the activities and the program?
   4. Did they want to engage in group sessions?
3. How did you feel about virtual S.M.I.L.E.?
   1. How did you like the format of a mix of group and individual activities?
   2. Did you have any issues accessing the program? Technology, wifi, etc.?
   3. How feasible was it to do the activities with your child?
   4. Did you feel that it was too much or too little work?
4. How did virtual S.M.I.L.E. meet or not meet your expectations?
5. How do you think virtual S.M.I.L.E. has impacted your child’s physical activity?
   1. Are they more or less active than before the program started?
   2. Are there any skills they have learned or anything that was missing?
6. What else might you have liked about virtual S.M.I.L.E.?
7. What else didn’t you like about virtual S.M.I.L.E.?
8. Would you enrol your child in virtual S.M.I.L.E. again?
   1. If in person (i.e. regular S.M.I.L.E.) wasn’t an option?
   2. If both were an option?
9. Is there anything else you want to tell us about your experience with virtual S.M.I.L.E.?
